# All-cause mortality of insulin plus dipeptidyl peptidase-4 inhibitors in persons with type 2 diabetes

**DOI:** 10.1186/s12902-018-0330-7

**Published:** 2019-01-05

**Authors:** Fu-Shun Yen, Jen-Huai Chiang, Chii-Min Hwu, Yu-Hsin Yen, Boniface J. Lin, James Cheng-Chung Wei, Chih-Cheng Hsu

**Affiliations:** 1 Dr. Yen’s Clinic, No.15, Shanying Road, Gueishan District, Taoyuan City, 33354 Taiwan; 2 Management Office for Health Data, China Medical University Hospital, No.91, Xueshi Road, North District, Taichung City, 40402 Taiwan; 3 College of Medicine, China Medical University, No.91, Xueshi Road, North District, Taichung City, 40402 Taiwan; 4 Section of Endocrinology and Metabolism, Department of Medicine, Taipei Veterans General Hospital, No. 201, Section 2 Shi-Pai Road, Chung-Cheng Building 11F, Room536, Taipei, 112 Taiwan; 5 Duke-NUS Medical School, 8 College Rd, Singapore, 169857 Singapore; 6 Dr. Lin Clinic, No.2, Section 2, Xinsheng South Road, Da’an District, Taipei City, 10650 Taiwan; 7 Division of Allergy, Immunology and Rheumatology, Chung Shan Medical University Hospital, No. 110, Section 1, Jianguo North Road, South District, Taichung City, 40201 Taiwan; 8 Institute of Population Health Sciences, National Health Research Institutes, No.35, Keyan Road, Zhunan Township, Miaoli County, 35053 Taiwan; 9 Department of Health Services Administration, China Medical University, No.91, Xueshi Road, North District, Taichung City, 40402 Taiwan; 10 Department of Family Medicine, Min-Sheng General Hospital, No. 168, Jingguo Road, Taoyuan, Taiwan

**Keywords:** All-cause mortality, Cohort study, Match

## Abstract

**Background:**

Dipeptidyl peptidase-4 (DPP-4) inhibitors could effectively reduce HbA_1C_ and postprandial hyperglycemia and could incur only minimal danger of hypoglycemia. Patients with uncontrolled diabetes might be treated by the complementary action of insulin plus DPP-4 inhibitors. Here, we compared the all-cause mortality risk between DPP-4 inhibitor users and nonusers with underlying insulin therapy.

**Methods:**

Using the population-based National Health Insurance Research Database of Taiwan, we conducted an 11-year retrospective cohort study. A total of 3120 patients undergoing insulin therapy for type 2 diabetes mellitus (T2DM) during 2000–2010 were enrolled. The overall incidence rates for all-cause mortality of 1560 DPP-4 inhibitor users and 1560 matched DPP-4 inhibitor nonusers were compared.

**Results:**

No significant difference was found in the baseline demographic and clinical variables of the two groups of patients. Median follow-up period for the matched cohort was 1.67 years. All-cause mortality was observed in 93 (6.0%) of 1560 DPP-4 inhibitor nonusers and 36 (2.3%) of 1560 DPP-4 users. The incidence rate of mortality was 11.72 for DPP-4 inhibitor users and 38.16 per 1000 person-years for DPP-4 inhibitor nonusers. After multivariate adjustment, DPP-4 inhibitor users ran a reduced mortality risk (adjusted hazard ratio 0.32, 95% CI 0.22–0.47; *p* < 0.0001) than did the nonusers.

**Conclusion:**

Risk of all-cause mortality may be reduced when using insulin plus DPP-4 inhibitors than when using insulin plus non–DPP-4 inhibitors.

## Background

Currently, diabetes mellitus (DM) incidence is increasing rather rapidly. Per the International Diabetes Federation, 425million adults are currently diagnosed with diabetes globally; by 2045, this number is expected to rise to 629 million. Approximately1in 11 adults has diabetes, and every six minutes a person dies from diabetes [1]. In addition, diabetes is a major source of the global burden of disease [2]. Therefore, new ways to prevent the occurrence of diabetes must be found. Patients with DM must receive optimal treatment. Upon diagnosis of DM, most patients were found to have a 50% reduction in their insulin secretion; this percentage continues to decline for 6 years after diagnosis. Eventually, most patients require insulin treatment, either alone or together with an oral agent [3]. However, Stark et al. reported that even with insulin treatment, approximately 70% of patients with diabetes fail to reach their glycemic goal [4]. Usually, basal insulin must be administered along with other oral antidiabetic drugs (OADs). Fonseca et al. conducted a meta-analysis and concluded that the early use of insulin with OADs could notably reduce glycated hemoglobin A1C (HbA_1C_) and that this approach resulted in relatively low rates of hypoglycemia [5]. However, the safety and outcomes of insulin combined with different OADs remained to be further investigated.

Following the ingestion of nutrients, the gastrointestinal duct secretes glucose-dependent insulinotropic peptide (GIP) as well as glucagon-like peptid-1(GLP-1), which exert a powerful effect on the secretion of insulin and glucagon from the pancreas. Subsequently, both GLP-1 and GIP are quickly degraded to inactive metabolites by dipeptidyl peptidase-4 (DPP-4). By inhibiting GLP-1and GIP degradation, inhibitors of DPP-4 can improve glycemic control. GLP-1 can decelerate gastric emptying and decrease postprandial hyperglycemia. GLP-1 reduces pancreatic alpha cell glucagon secretion in hyperglycemic states. GIP can increase glucagon secretion during hypoglycemia [6]. Because DPP-4 inhibitors effectively lower postprandial glucose levels and decrease the probability of hypoglycemia, DPP4-inhibitors could harmonize the safety and action of insulin treatment. Therefore, combined treatment using DPP-4 inhibitors plus insulin is a potentially effective treatment approach for patients with poor diabetes control.

Insulin coupled with vildagliptin has been reported to significantly decrease HbA_1C_ compared with insulin monotherapy and to trigger fewer and less severe hypoglycemic events [7]; however, these results were from short-term randomized control trials with no long-term clinical outcomes. Therefore, in this retrospective cohort study, we assessed the risk of all-cause mortality for a treatment regimen that combined insulin and DPP-4 inhibitors.

## Methods

### Data source

Implemented in 1995, the National Health Insurance program is a mandatory, single-payer program that currently covers 99% of the 23 million residents of Taiwan [8]. Data for this study were extracted from the National Health Insurance Research Database (NHIRD). The NHIRD holds information regarding inpatient claims, ambulatory claims, medical institutions, drug prescriptions, and other data. The Longitudinal Health Insurance Database 2000 (LHID2000) is a subset of the NHIRD that holds the primary insurance data, from the year 2000, of 1,000,000 randomly sampled beneficiaries. This dataset includes data on sex, date of birth, procedures, orders, and diagnosis codes based on the International Classification of Diseases, Ninth Revision, Clinical Modifications (ICD-9-CM). For the present investigation, all patient data were derived from the LHID2000. Because all care providers’ and patients’ identifying information is encrypted in the LHID2000, no patient could be identified using the collected data. Thus, the usual requirement to receive consent forms from the participants was waived for this study, which was approved by The Research Ethics Committee of China Medical University and Hospital (CMUH104-REC2–115).

### Study population

This retrospective cohort study used Taiwanese NHIRD administrative data from January 1, 1997, to December 31, 2010. For the period 2000–2010, newly diagnosed type 2 patients with diabetes (T2DM; ICD-9-CM: 250.x) in the age range of 18–100 years were selected. Only those patients diagnosed with T2DM at least twice in outpatient claims or at least once in inpatient claims were included to ensure diagnostic accuracy. Each of these patients had received insulin therapy after being diagnosed with T2DM. Criteria for rejecting patients were diagnosis of type 1 diabetes (250.1x), rheumatic heart failure (HF, 398.91), stroke (430–438), peripheral arterial occlusive disease (443.9440–444), or HF (428) before the index date of the study.

### DPP-4 inhibitors

Patients who had received DPP-4 inhibitors before or after insulin treatment were recruited. The date of concurrent use of insulin plus DPP-4 inhibitors was deemed the index date. For DPP-4 inhibitor nonusers, index dates were randomly assigned corresponding to the index date of the paired DPP-4 user. The DPP-4 inhibitors prescribed were saxagliptin, sitagliptin, vildagliptin, and linagliptin.

### Primary outcome and causes of death

All-cause mortality was the primary outcome of this study. The observation period spanned from the index date and proceeded to the date of death, date of withdrawal from the NHI program, or December 31, 2010, whichever came first. We also assessed the last primary diagnosis of discharge 3 months before death to search for the causes of death [9]. Per the Standardized Definitions for End Point Events in Cardiovascular Trials [10], the causes of cardiovascular (CV) death include the following: 1. ischemic heart disease [coronary artery disease, 410–414, 429.2; myocardial infarction (MI), 410, 411.0, 412, and 429.79]. 2. Sudden cardiac death (cardiac arrhythmia, 427; sudden cardiac arrest, V12.53**)**. 3. HF (398.91, 402.01, 402.11, 402.91, and 428). 4. Stroke (430–438). 5. CV procedures (668.1 and 997.1). 6. CV hemorrhage (cardiac tamponade, 423.3; aortic aneurysm and dissection, 441). 7. Other CV causes (arterial embolism and thrombosis, 444). Regarding non-CV causes of death, we assessed cancers (140–208), infection (001–139, 320, 321,326, 421, 460–466, 480–487,510, 513, 551,567, 590, 599,680–686, 711,730), nephropathy (580–589), digestive diseases (520–579, excluding 551), respiratory diseases (518.81, 518.82, 518.85, 786.09, 799.1), accidents (800–949), and suicide (950–959). The cases for which we could not get the last main diagnosis 3 months before death were defined as undetermined causes of death.

### Demographics and comorbidities

Risk factor–related comorbidities analyzed in this study include ischemic heart disease (411–414), dyslipidemia (272, A code: A182) and hypertension (401–405, A code: A260 and A269). The conditions were deemed comorbid, if a patient was diagnosed in at least two of outpatient records or at least once in inpatient records, before the initial diagnosis of T2DM.

In our patient group, we also observed the prescription of non–DPP-4-inhibitors, non-insulin antidiabetic drugs following T2DM diagnosis, for example, biguanides (metformin, buformin), sulfonylureas (chlorpropamide, acetohexamide, glibornuride, glibenclamide, gliclazide, glipizide, gliquidone, glimepiride, tolbutamide, and tolazamide), alpha-glucosidase inhibitors and thiazolidinediones (rosiglitazone, pioglitazone).

### Statistical analysis

We compared the baseline characteristics, comorbidities, and other medications between users and nonusers of DPP-4 inhibitors. We used one-to-one propensity scores to match the age (per 5 years), sex, drugs used, comorbidities, initial year of diabetic diagnosis, and index year [11]. The propensity scores of all patients were estimated through non-parsimonious multivariate logistic regression, wherein intake of DPP-4 inhibitors and 13 clinically relevant covariates (baseline characteristics) as the dependent variable and the independent variables, respectively. Matched pairs were constructed using a nearest-neighbor algorithm, with 0_._995–1_._0 as the perfect proportion [12]. Differences in the categorical and continuous variables of the two groups were analyzed through chi-square test and Student t-test, respectively; whereas a Cox proportional hazard model was employed to determine the hazard ratios (HRs) and 95% confidence intervals (95% CI). Survival probability was derived using log-rank tests and Kaplan–Meier estimations. *P* < 0.05 was deemed significant. All analyses were performed using SAS 9.4 (SAS Institute Inc., Cary, NC).

## Results

**Fig. 1:**
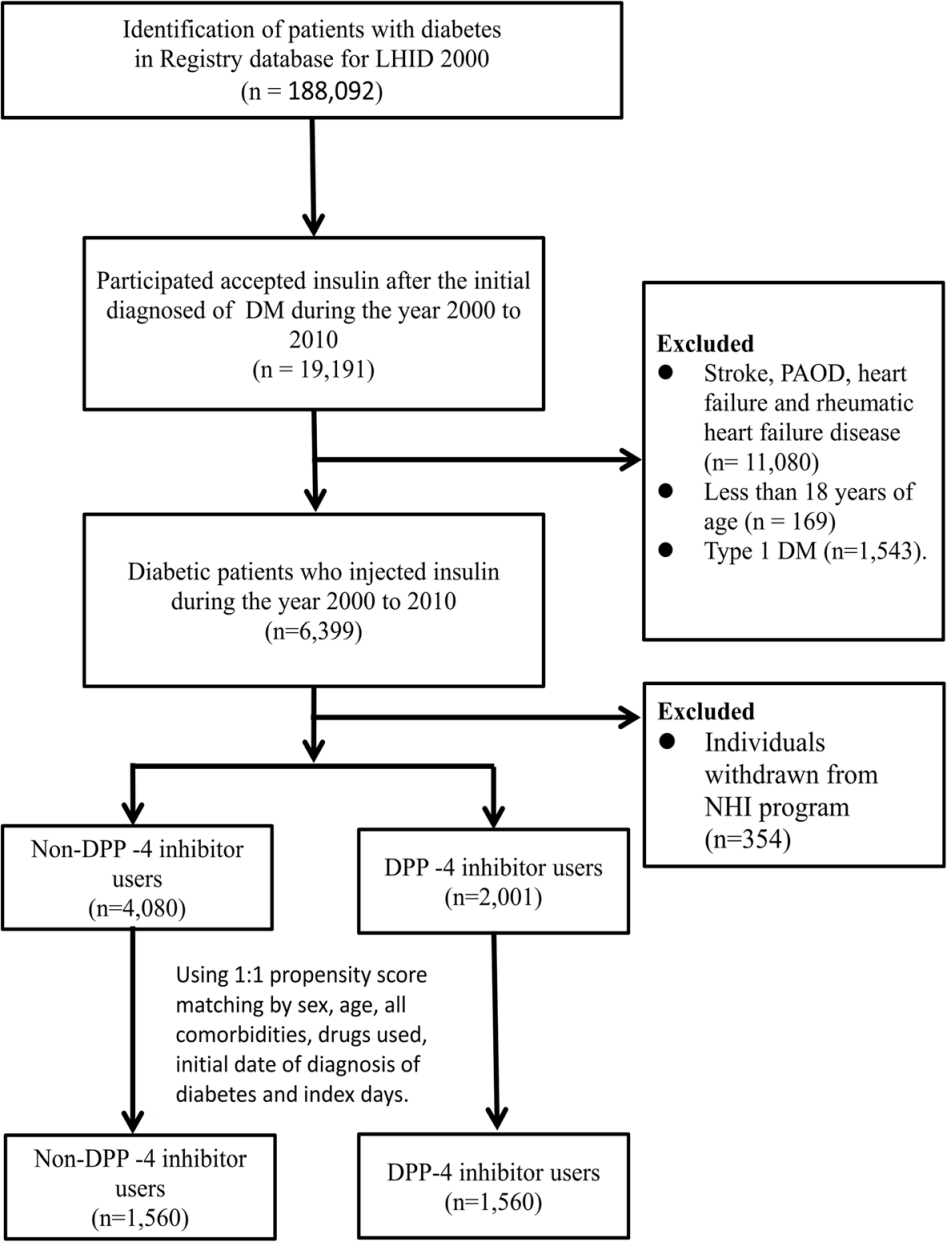
Flow chart showing study design

The selection criteria described earlier yielded 3120 patients with diabetes (i.e., 1560 for each cohort) (Fig. 1). Table 1 shows their demographics. This group had more men than women. After propensity score matching, no variable differed significantly between DPP-4 inhibitor nonusers and users. The mean ages (standard deviation) of DPP-4 inhibitor nonusers and users were 49.23 (11.77) and 49.21(11) years, respectively, and the corresponding mean follow-up periods were 1.56 (1.61) and 1.97 (1.74) years.

**Table 1:** Demographics and comorbidities in the study group and the control group

Variables	Accepted DPP-4 inhibitors				*p*-value
	No		Yes		
	(*n* = 1560)		(*n* = 1560)		
	N	%	n	%	
Sex					0.8545^a^
Female	614	39.36	609	39.04	
Male	946	60.64	951	60.96	
Age, years					0.0919^a^
18–39 years	344	22.05	314	20.13	
40–64 years	1065	68.27	1119	71.73	
more than 65 years	151	9.68	127	8.14	
Age, Mean (SD)^b^	49.23 (11.77)		49.21 (11.00)		0.9573^b^
Comorbidity					
Ischemic heart disease	133	8.53	136	8.72	0.8483^a^
Hypertension	512	32.82	509	32.63	0.9089^a^
Dyslipidemia	327	20.96	327	20.96	0.99^a^
Other drugs					
Biguanides	1546	99.10	1546	99.10	0.99^a^
Sulfonylureas	1510	96.79	1494	95.77	0.13^a^
Thiazolidinediones	680	43.59	680	43.59	0.99^a^
Alpha glucosidase inhibitors	628	40.26	666	42.69	0.1673^a^
Duration between initial date of diagnosis of diabetes and index days (mean, median)	2531 (2530)		2504 (2562)		0.5059^b^

^a^Chi-square test

^b^Two sample t-test

**Fig. 2:**
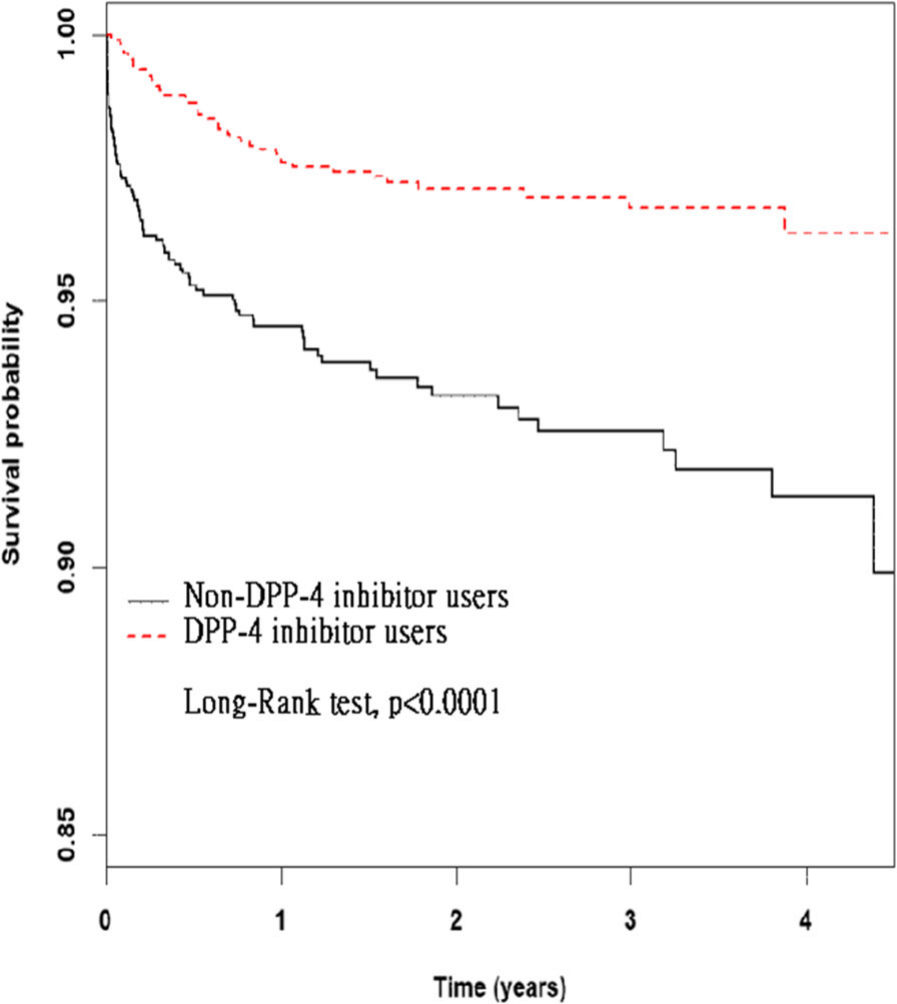
Survival probability in the insulin with DPP-4 inhibitors and insulin without DPP-4 inhibitors group, obtained through Kaplan–Meier analysis

Among DPP-4 inhibitor nonusers and users, the mortality rates were 38.16 and 11.72 per 1000 person-years, respectively, while the adjusted HR of mortality of users relative to nonusers was 0.32 (95% CI: 0.22–0.47, *P* < 0.0001; Table 2). Moreover, the Kaplan–Meier analysis (Fig. 2) revealed that DPP-4 inhibitor users had a higher survival probability than did nonusers (log-rank test, *P* < 0.0001).

**Table 2:** Risk of mortality for patients with type 2 diabetes receiving insulin treatment

Characteristics	Mortality no. (*n* = 129)	Crude			Adjusted		
		HR	(95% CI)	*P*-value	HR	(95% CI)	*P*-value
DPP-4 inhibitors							
No	93	1.00	reference		1.00	reference	
Yes	36	0.33	(0.22–0.48)	<.0001	0.32	(0.22–0.47)	<.0001
Sex							
Female	34	1.00	reference		1.00	reference	
Male	95	1.86	(1.25–2.75)	0.002	1.89	(1.28–2.8)	0.0015
Age, years							
18–39 years	12	1.00	reference		1.00	reference	
40–64 years	94	2.51	(1.38–4.58)	0.0027	2.80	(1.53–5.14)	0.0009
≥ 65 years	23	5.37	(2.67–10.81)	<.0001	6.26	(3.04–12.87)	<.0001
Comorbidity							
Ischemic heart disease	17	1.63	(0.98–2.72)	0.0598	1.71	(0.98–2.98)	0.0601
Hypertension	38	0.88	(0.6–1.28)	0.4979	0.68	(0.45–1.04)	0.0774
Dyslipidemia	21	0.77	(0.48–1.22)	0.2666	0.76	(0.46–1.23)	0.2595
Other drugs							
Biguanides	128	1.10	(0.15–7.86)	0.9253	0.91	(0.11–7.2)	0.9263
Sulfonylureas	127	2.18	(0.54–8.8)	0.2752	2.38	(0.55–10.37)	0.2473
Thiazolidinediones	41	0.52	(0.36–0.75)	0.0005	0.55	(0.38–0.81)	0.0022
Alpha glucosidase inhibitors	41	0.58	(0.4–0.84)	0.0039	0.67	(0.46–0.98)	0.0415

*HR* hazard ratio, *CI* confidence interval

Adjusted HR: adjusted for use of DPP-4 inhibitors, age, gender, comorbidity and other drugs used in Cox proportional hazards regression

The major identifiable causes of death of DPP-4 inhibitors users were as follows: 0 CV deaths, 31 (1.992%) non-CV deaths, and 5 (0.32%) undetermined cases. The identifiable causes of death of nonusers were as follows: 3 (0.19%) CV deaths, 78 (5.00%) non-CV deaths, and 12 (0.77%) undetermined cases.

## Discussion

Our analysis confirmed that treatment using insulin plus DPP-4 inhibitors carried a lower risk of all-cause mortality than did treatment using insulin plus non–DPP-4 inhibitors.

Some randomized clinical trials have explored the efficacy and safety of co-administering DPP-4 inhibitors and insulin to T2DM patients. Fonseca et al. used vildagliptin [7], and Hong et al. used sitagliptin [13]; both reported significantly lower risk of hypoglycemia relative to a placebo. Lukashevich et al. [14], Kothnyetal. [15], and Kozlovskiet al. used vildagliptin [16]; Barnett et al. used saxagliptin [17]; Arnolds et al. used sitagliptin [18]; Yki-Jarvinen et al. used linagliptin [19]; all of these investigators have reported neutral effects on hypoglycemia. Visbøll et al. used sitagliptin and reported significantly higher risk of hypoglycemia [20]. These clinical trials all demonstrated significant lowering of hemoglobulin A1C (HBA_1C_) and fasting plasma glucose levels, but with variable results for hypoglycemia; the reasons for such variable results regarding hypoglycemia might relate to their different patient groups and study designs. However, all these reports were from short-term and small-series clinical studies; no long-term outcomes have been noted.

Nevertheless, three recent large safety trials involving DPP-4 inhibitors have reported that these inhibitors do not raise the risks of major adverse CV events and mortality [21–23]. Pasquel et al. found that, for patients with T2DM, the treatment regimen of sitagliptin plus basal insulin is as safe and as effective as is the basal-bolus regimen [24]. In a post-hoc analysis, Zinman et al. investigated the CV safety of linagliptin as an insulin add-on in patients with T2DM [25] and found a neutral effect on major CV events (linagliptin 27 vs. placebo 24 events) and overall mortality (8 deaths in 811 patients vs. 8 deaths in 802 patients). The event rates of total mortality in this analysis might be too small to analyze. Nevertheless, our study confirmed that insulin plus DPP-4 inhibitors carries a reduced mortality risk (adjusted HR = 0.32, *P* < 0.0001). Our analysis differs from that of Zinmen et al. likely because of the differences in the characteristics of the patient populations as well as the differences in the insulin plus DPP-4 inhibitor regimens; furthermore, our study was a large series that entailed numerous incidence rates. Similarly, the differences in mortality between our study and the three afore mentioned may be attributable to all our patients receiving insulin treatment, unlike in the trials. The reduced mortality identified in this study may be attributable to the following: (1) DPP-4 inhibitors effectively decreased blood glucose level and HbA_1C_, with low hypoglycemia risk and no increase in body weight [6]. (2) DPP-4 inhibitors diminished glucose excursion as well as hindered carotid atherosclerotic processes [26]. (3) The DPP-4 inhibitors increased stromal cell–derived factor 1 and recruited endothelial progenitor cells to the ischemic tissue, resulting in myocardial protection [27]. DPP-4inhibitors also enhanced the endothelial function, slowed down atherogenesis, diminished ischemia and reperfusion injury, prevented left ventricular hypertrophy and remodeling, and reduced inflammatory markers [4]. As reported in the literature, DPP-4 inhibitors could increase adiponectin levels and bring about modest reductions in lipidemia and blood pressure [28].

There were 0 CV death in the insulin plus DPP-4 inhibitors users and only 3 CV deaths in the non-users in our study. The low number of CV death might be because we had excluded the high-risk patients, such as those with heart failure, stroke or peripheral arterial occlusive disease, from the study cohort. The mean follow-up time in our study is less than 2 years, which also might lead to few cases of CV death.

Consistent with nationwide surveys in Taiwan [29], our study also found that male and elderly patients have relatively high mortality rates. In addition, our study showed that pioglitazone users had a relatively low mortality rate. Charbonnel et al.’s analysis of the PROactive trial demonstrated that pioglitazone plus insulin regimen yielded sustained improvements in glycemic control and reduced insulin doses, but it increased edema, hypoglycemia, and HF rates, with a non-significant 15% reduction in risk in the major secondary endpoint (i.e., a composite of nonfatal MI, stroke, and death) [30]. Additional rigorous analyses are essential to elucidate these results in real-world practice.

In conducting the observational studies there are several methodological issues should be addressed, such as selection bias, immortal time bias and confounding by indication. Because our database is from the National Health Insurance program, which covers 99% of the whole residents of Taiwan, selection bias could be avoided. The first date of concurrent use of insulin and DPP-4 inhibitors was defined as the index date; so, we didn’t create a follow-up period (i.e., immortal time window) within which the investigated outcomes could not happen; the chance of immortal time bias is low. The prescription of medication in the clinical practice is influenced by indications, contraindications, side effects and the preferences of patients and doctors. This might lead to the bias of “cofounding by indication”; however, we performed a 1:1 propensity score match to balance the 13 clinically relevant covariates between the users and non-users. We believe the two study groups were comparable and the bias of cofounding by indication could be minimized as much as possible.

The present study has several strengths. First, as far as we know, this is the first study to disclose that an insulin plus DPP-4 inhibitor regimen could reduce mortality more than an insulin plus non–DPP-4 inhibitor regimen. Second, this is a large-series population-based study; the dataset is greatly representative of Taiwan’s population. Third, sex, age, comorbidities*,* other antidiabetic drugs, and DM duration were all matched through propensity scoring to eliminate confounding factors.

However, the present study carries some limitations. Firstly, in the NHIRD, we could not obtain any data relating to the prescribed insulin dose; this is relevant because dosage might influence the analytic results. Secondly, the NHIRD does not include data on life styles, smoking habits, body weights, and economic conditions, all of which could influence mortality risks. Third, the dataset lacks biochemical blood tests, which can further express the conditions of patients under treatment. Finally, any cohort study is always subject to some inevitable biases; a randomized control trial should be conducted to affirm our results.

## Conclusions

Insulin administered with DPP-4 inhibitors was able to reduce mortality and increase survival probability relative to the use of non–DPP-4 inhibitors. However, more studies are necessary to optimize the use of these DPP-4 inhibitors in real-world practice.

## Abbreviations

CI: Confidence interval
CV: Cardiovascular
DM: Diabetes Mellitus
DPP-4inhibitors: Dipeptidyl peptidase-4 inhibitors
GIP: Glucose-dependent insulinotropic peptide
GLP-1: Glucagon-like peptid-1
HbA_1C_: Hemoglobin A1C
HF: Heart failure
HRs: Hazard ratios
ICD-9-CM: International Classification of Diseases, Ninth Revision, Clinical Modifications
LHID: Longitudinal Health Insurance Database
MI: Myocardial infarction
NHIRD: National Health Insurance research database
OADs: Oral antidiabetic drugs
T2DM: Type 2 diabetes mellitus
